# Preliminary Investigation of Focused Ultrasound-Facilitated Drug Delivery for the Treatment of Leptomeningeal Metastases

**DOI:** 10.1038/s41598-018-27335-y

**Published:** 2018-06-13

**Authors:** Meaghan A. O’Reilly, Tricia Chinnery, My-Linh Yee, Sheng-Kai Wu, Kullervo Hynynen, Robert S. Kerbel, Gregory J. Czarnota, Kathleen I. Pritchard, Arjun Sahgal

**Affiliations:** 1 Physical Sciences Platform, Sunnybrook Research Institute, Toronto, Canada; 2 Department of Medical Biophysics, University of Toronto, Toronto, Canada; 3 Institute of Biomaterials and Biomedical Engineering, University of Toronto, Toronto, Canada; 4 Biological Sciences Platform, Sunnybrook Research Institute, Toronto, Canada; 5 Radiation Oncology, Sunnybrook Health Sciences Centre, Toronto, Canada; 6 Department of Radiation Oncology, University of Toronto, Toronto, Canada; 7 Evaluative Clinical Sciences, Sunnybrook Health Sciences Centre, Toronto, Canada; 8 Medical Oncology, Sunnybrook Health Sciences Centre, Toronto, Canada; 9 Department of Medicine, University of Toronto, Toronto, Canada

## Abstract

Leptomeningeal metastases (LM) are a serious complication of cancer in the central nervous system (CNS) and are diagnosed in approximately 5% of patients with solid tumors. Effective treatment using systemically administered therapeutics is hindered by the barriers of the CNS. Ultrasound can mediate delivery of drugs through these barriers. The goal of this study was to test the feasibility of using ultrasound-mediated drug delivery to improve the treatment of LM. LM was induced in the spinal cord of athymic rats by injecting HER2-expressing breast cancer cells into the subarachnoid space of the thoracic spine. Animals were divided into three groups: no treatment (n = 5), trastuzumab only (n = 6) or trastuzumab + focused ultrasound + microbubbles (FUS + MBs) (n = 7). Animals in groups 2 and 3 were treated weekly with intravenous trastuzumab +/− FUS + MBs for three weeks. Suppression in tumor growth was qualitatively observed by MRI in the group receiving ultrasound, and was confirmed by a significant difference in the tumor volume measured from the histology data (25 ± 17 mm^3^ vs 8 ± 5 mm^3^, p = 0.04 in the trastuzumab-only vs trastuzumab + FUS + MBs). This pilot study demonstrates the potential of ultrasound-mediated drug delivery as a novel treatment for LM. Future studies will extend this work to larger cohorts and the investigation of LM arising from other cancers.

## Introduction

Leptomeningeal metastases (LM) refers to metastatic involvement of the meninges that line the brain and spinal cord, and are diagnosed in approximately 5% of patients with solid tumors^1^. Breast cancer accounts for the greatest number of cases of LM^1^, and autopsy reports suggest that the true incidence in this population may be greater than 16%^2^. Further, the incidence is increasing as better systemic control improves patient survival and the methods for diagnosing LM improve^3^. LM causes neurological symptoms due to compression and infiltration of the brain and spinal cord, and obstruction of normal cerebrospinal fluid (CSF) flow^1^. The prognosis once LM is diagnosed is extremely poor, with a median survival of approximately 4.5 months^4^.

There are several factors that prevent effective treatment of LM. First, LM is an inherently multifocal disease. Cancer cells in the CSF are transported throughout the subarachnoid space and seed the meninges^1^, resulting in widely distributed lesions that can cover significant portions of the brain and spinal cord. Therefore, surgery is not a curative option and has no therapeutic role in the management of LM. Radiation therapy has been the treatment of choice in these patients but limited to a palliative role. For example, the current standard of care for LM in the brain is palliative whole brain radiation, which can stabilize neurological symptoms temporarily^5,6^. Similarly, in the spine the current treatment of LM is palliative radiation. However, unlike the brain, the fields of spinal radiation are limited to areas of gross disease as opposed to treating the entire spinal axis due to toxicity. Therefore, systemic therapy is attractive for these patients as ideally the aim is to treat not only gross disease but the microscopic burden throughout the spine. However, LM have been shown to respond poorly to systemic therapy whether intravenous or intrathecal.

The lack of response in the CNS to chemo- and immunotherapy agents is largely related to the blood-brain barrier (BBB) and blood-spinal cord barrier (BSCB) that restrict the passage to small (<500 Da) molecules with high lipid solubility^7^. These barriers prevent intravenously administered anti-cancer agents from accumulating in the tumor in therapeutically relevant quantities. While the tumor cells in the CSF can be targeted using intrathecal chemotherapy^8,9^, once bulk deposits are more than a few cells thick, intrathecal agents also cannot effectively penetrate the tumors.

One method to circumvent the BBB and BSCB is through the use of focused ultrasound (FUS). It has been shown that ultrasound combined with circulating ultrasound contrast agents (micron-sized stabilized bubbles known as ‘microbubbles’) can be used to temporarily and reversibly open the BBB to allow drugs to reach the brain^10^. This occurs because the microbubbles (MBs), which are injected intravenously, oscillate in the ultrasound field and stimulate the blood vessel walls. In preclinical brain studies, this technique has been shown to facilitate the delivery of therapeutics, ranging from small molecule chemotherapeutics^11–13^, to antibodies^14,15^, gene-delivery vectors^16,17^ and stem cells^18^, and has been highly successful in delivering chemotherapy to brain tumors, with complete tumor eradication observed in some glioma-bearing rats^13^. Recently, we^19^ and others^20^ have shown in a small animal model that the BSCB can be influenced using similar methods, although drug delivery through the BSCB has never been tested in a disease model. We hypothesize that FUS-mediated delivery of therapeutics through the BSCB as a treatment for LM in the spinal cord has the potential to significantly improve outcome.

Here we test this hypothesis is a small pilot study in athymic nude rats bearing bulk LM lesions in the spinal cord.

## Results

### MRI findings

**Figure 1:**
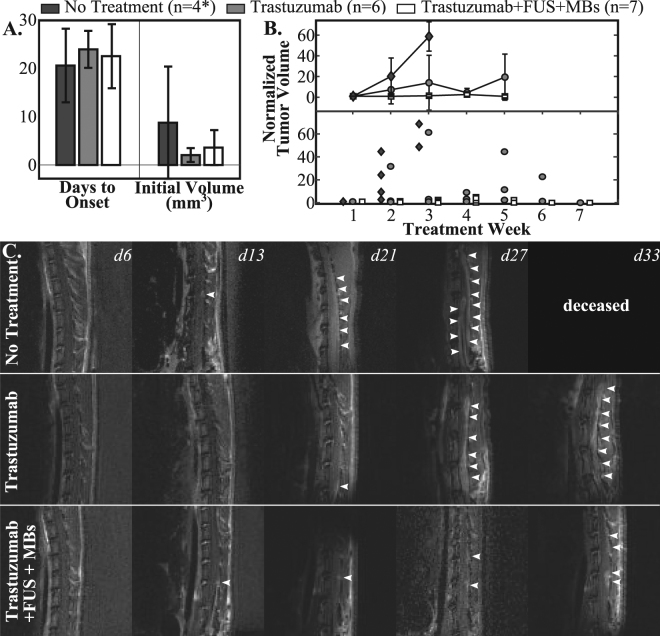
(**A**) Mean days to onset and initial volume measured by MRI. (**B**) Weekly normalized tumor volume (top: mean ± S.D., bottom: individual data points), normalized to the initial tumor volume. (**C**) Example image series (contrast-enhanced T_1_weighted MRI) for each of the three groups. The white arrowheads show the tumor regions. *Data from the No Treatment group shown in A and B excludes one animal.

Enhancing bulk LM deposits were first detected by MRI between d13 and d30 post-injection of the tumor cells (median = d23). There were no differences between any of the groups for mean time to tumor detection or the initial tumor volume at detection (Fig. 1A). The data shown in Fig. 1 excludes one animal from the untreated control group. This exclusion was due to venous access issues and consequent imaging issues. In most cases the use of intraperitoneal administration of the MRI contrast agent was successful when intravenous administration failed. However, in one animal an adequate contrast image could not be obtained for one of the time points making accurate estimation of the tumor onset time and initial volume impossible and necessitating the exclusion of the animal from this portion of the analysis. Figure 1B shows the tumor volume, as assessed by contrast MRI and normalized to the initial volume, at the different imaging time-points. The average weekly volumes are shown in the top plot, with the individual data points shown in the bottom plot. Although the normalized tumor volume in the trastuzumab-only group was consistently higher than in the trastuzumab + FUS + MBs group, no statistical differences (t-test, equal variance) were observed. One animal in the trastuzumab only group died one week following the first treatment, before imaging to assess the tumor growth could be performed. The tumors appear to grow the fastest in the untreated control group, which is consistent with the short survival time observed in these animals (see below). However, the small group size prevents meaningful comparisons. In particular, by week 3 only two animals remained in the untreated control group, preventing any statistical comparisons with this group. In Fig. 1C, representative contrast MRI panels are shown for one animal in each of the three groups. In the animal receiving trastuzumab the tumor rapidly grows and by d33 there is extensive involvement of the spinal cord. The untreated control had large tumor burden by d27 and did not survive to the d33 imaging time point. In contrast, in the animal receiving trastuzumab + FUS + MBs the tumor progression is more controlled.

### Overall Survival

**Figure 2:**
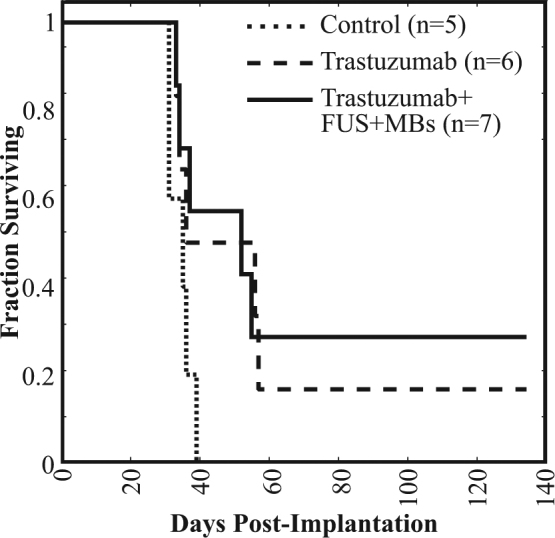
Survival curves for the three groups.

The apparent improvement in tumor control did not lead to large improvements in overall survival (Fig. 2) between Groups 2 and 3. Although both performed better than Group 1, statistical significance was not observed. The reason for this was believed to be due to systemic disease involvement. Table 1 summarizes the study endpoints reached. Nine of the 18 animals reached the motor-dysfunction endpoint. Additionally, 1 animal was sacrificed due to self-mutilation of the tail, which cannot be ruled out as a neurological symptom related to tumor growth. However, five animals were lost to significant (>20%) weight loss, and at necropsy these animals appeared to have some involvement of the lungs.

**Table 1:** Summary of study endpoints reached.

	Weight Loss	Self-Mutilation	Motor Dysfunction (Hind Limb)	Motor Dysfunction (Front Limb)	Study End
Control (n = 5)	2		3		
Trastuzumab (n = 6)	2		2	1	1
Trastuzumab + FUS + MB (n = 7)	1	1	2	1	2

### Histology Findings

**Figure 3:**
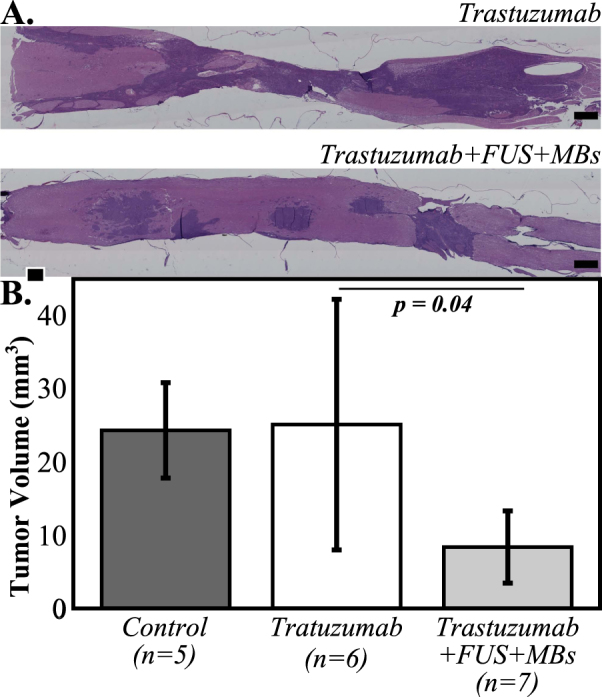
(**A**) Representative histology sections from animals in the trastuzumab only and trastuzumab + FUS + MBs groups illustrating reduced tumor burden in the animal treated with trastuzumab + FUS + MBs compared to that receiving the drug alone. (**B**) Tumor volume based on quantification of the histology data for the three groups. Error bars indicate one standard deviation.

That some of the attrition may be due to factors other than LM tumor burden is supported by the histology data (Fig. 3). Analysis of these data confirms that the tumor burden in the trastuzumab only group was significantly greater (p = 0.04) than that of the trastuzumab + FUS + MBs group (mean tumor volume = 25 ± 17 mm^3^ vs 8 ± 5 mm^3^), and near significance (p = 0.06) was observed between the trastuzumab + FUS + MBs group and the control group (24 ± 7 mm^3^).

There were three long-term survivors, one in the trastuzumab only group and two in the trastuzumab + FUS + MBs group. These animals had very low tumor volumes (2.3, 4.5 and 0 mm^3^). One animal from each group was removed from the study at 135 days post-implantation. The remaining animal (trastuzumab + FUS + MBs) was inadvertently allowed to survive until d180. This animal had no tumor cells detectable on histology. There is some concern that had the other animals survived longer, improvements might also have been seen in their tumors. However, if we analyze the hypothetical worst-case scenario where the animal from the trastuzumab-only group (tumor volume 2.3 mm^3^) achieves zero tumor volume given a longer survival, but no change is seen in the FUS + MBs group, statistical significance (p = 0.049) is still achieved.

## Discussion

This preliminary study demonstrates the potential of ultrasound-mediated drug delivery for the improved treatment of LM. We acknowledge the group size was small for this pilot work and no improvement in overall survival was observed. However, the MRI and histology data show promise for this therapy. In this study the tumor cell injections were performed one week following the catheter implantation surgery. Although injection into the subarachnoid space was expected to confine the cells to the protected CNS space, the surgical site, particularly if was still healing at the time of cell injection, may have provided an entry point for tumor cells to exit the CNS and spread systemically. To minimize the risk of systemic involvement, in future studies the animals will have a longer post-surgical recovery time before injection of the tumor cells. This, combined with larger group sizes, will hopefully lead to the improved tumor control translating to an improvement in survival.

In addition to the small sample size, this study has several other limitations. First, the treatments only targeted the bulk metastases and did not address the free cells in the CSF compartment. Ultimately this treatment would have to be combined with a method, such as intrathecal drug administration, to kill the cells in the CSF. Further, we did not quantify drug delivery to the tumors, and because of the high cost of this animal model there were a limited number of control groups. We have therefore assumed that the improvements seen are the result of enhanced drug delivery, but could also be due to the ultrasound exposure promoting an immune response^21^. A previous study using FUS + MBs to promote the delivery of trastuzumab to HER2+ brain tumors in rats found little difference between no treatment, FUS + MBs-only and trastuzumab-only groups. However, a significant improvement in outcomes for FUS + MBs + trastuzumab^22^ was observed, which supports our assumption that the suppression of the tumor growth is related to improved drug delivery. In future studies we will expand the control groups to examine the effects of FUS and FUS + MBs alone, and also to compare this approach to intrathecal administration of trastuzumab.

Other limitations in this study include the relatively large MRI voxel size (0.33 × 0.33 × 0.5 mm^3^), which makes accurate estimation of the tumor volume from the MRI data challenging for thin LM deposits. This could account for the lack of statistically significant differences observed by MRI. In future studies optimization of the imaging protocol and a spine specific imaging coil might allow for sufficient SNR to be achieved with a smaller voxel size without leading to prohibitively long imaging times.

Finally, the experiments in this study were performed in two cohorts. We observed a learning curve in the surgical procedure that resulted in a faster surgical recovery in the second cohort and more animals reaching the desired endpoint of motor dysfunction, rather than e.g. weight loss. The random assignment of the animals across the three groups in each cohort should eliminate any bias from this. However, others wishing to work with this animal model should be aware of this learning curve. We found instruction from an orthopaedic surgery resident in our institution’s spine program with prior research experience in rat laminectomy procedures to be invaluable, as well as extensive practice *ex vivo* on tissue obtained at the completion of other studies.

In this study, we chose to investigate a breast cancer subtype. In the future we would like to expand this work to investigate other breast cancer subtypes, as well as leukemias and lymphomas where LM may be present without systemic disease^23^. Leukemias and lymphomas have the highest incidence of LM, but are a smaller patient population and do not account for as many cases as solid tumors^23^. Ultrasound can mediate the delivery of a wide range of molecules and thus the specific therapeutic delivered could be tailored to the cancer type being studied.

Implementation of ultrasound + MB-mediated drug delivery to the spinal cord at clinical scale will rely on the development of methods to focus the ultrasound beams through the posterior elements of the spinal column. Methods have been developed for transcranial^24,25^ and transcostal^26^ focusing of therapeutic ultrasound beams, and these approaches can be adapted for the spinal geometry. In combination with existing methods for the brain, the advancement of drug delivery for the spinal cord could yield a non-ionizing treatment capable of targeting leptomeningeal metastases throughout the entire neuroaxis.

## Materials and Methods

**Figure 4:**
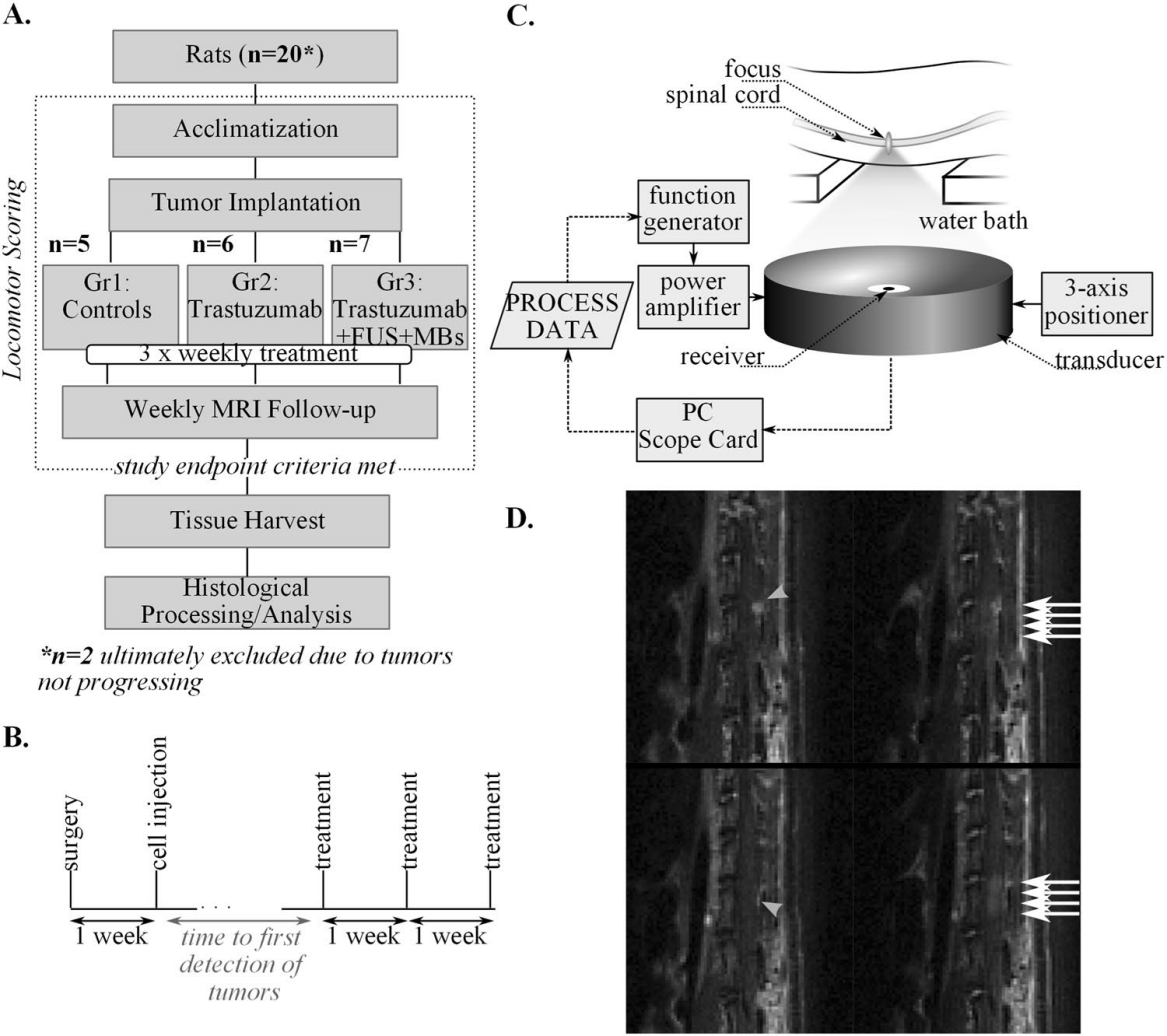
(**A**,**B**) Study design and treatment timeline; (**C**) Illustration of the FUS experimental setup; (**D**) Example T_1_weighted sagittal MR images showing the tumor location (grey arrowheads) before FUS + MB and the enhancement of the cord indicating BSCB opening (white arrows) post-FUS + MB.

All animal procedures were carried out with the prior approval of the Sunnybrook institutional animal care committee, and in accordance with the guidelines of the Canadian Council on Animal Care. A flowchart overviewing the study design is shown in Fig. 4A.

### Cell Culture

HER2-expressing human breast cancer cells (MDA-MB-231-H2N^27^) were used in this study. H2N is a variant of the highly tumorigenic MDA-MB-231 cell line by her2/erbB-2 gene transfection^27^. The cells were cultured in Dulbecco’s Modified Eagle’s Medium (DMEM) supplemented with 10% heat-inactivated fetal bovine serum (FBS, Wisent, St. Bruno, QC Canada) in 10 cm tissue culture plates in a 5% CO_2_-containing incubator at 37 °C. Cell number and viability were calculated using a hemocytometer and trypan blue exclusion. HER2 expression was confirmed using immunocytochemistry. MDA-MB-231-H2N cells were treated with an Allophycocyanin (APC) mouse anti-human HER-2/neu antibody (BD Biosciences, San Jose, CA, USA) overnight and then washed with PBS with 0.1% sodium azide. Cells were fixed with 1% paraformaldehyde and mounted with DAPI (Vector Laboratories, Burlingame, CA USA). Fluorescent images captured with a Zeiss AxioImager microscope confirmed HER2 expression.

### Tumor Implantation

LM was induced in the spinal cord of athymic rats (female, n = 20, ~200 g) using the method described by Janczewski *et al*.^28^. In brief, the animals were anesthetized using isofluorane (2%) and a laminectomy was performed at L4. Sterile PE-10 tubing, pre-flushed with saline, was inserted into the subarachnoid space and directed 2 cm cephalad. The proximal end of the tubing was tunneled subcutaneously to exit near the neck. The surgical incision was closed using sutures and the animals were given an analgesic (buprenorphine) and a non-steroidal anti-inflammatory (meloxicam) and were allowed to recover. In most animals, unilateral hind-limb dysfunction was observed post-treatment which was most likely due to irritation of the cord during the catheterization. This was fully resolved in 17/20 animals by 1 week post-surgery, with the remaining 3 animals showing continuing improvement. All hind-limb function was normal by 15 days post-surgery. Tumor cells (3 × 10^5^ cells suspended in 60 μl PBS) were injected via the catheter 7 days post-surgery. Figure 4B shows the relative timing of the surgery, cell injection and treatments.

### Magnetic Resonance Imaging

The animals were imaged on a 7 T MRI (BioSpec 70/30 USR; Bruker, Billerica, Mass) weekly beginning day 6 post-implantation. Animals were anesthetized with isofluorane at 2%. Sagittal contrast-enhanced (CE) T_1_-weighted images (RARE, TE = 10 ms, TR = 500 ms, matrix = 150 × 150, in-plane voxel dimensions = 0.33 × 0.33 mm^2^, slice thickness = 0.5 mm, Rare Factor = 2, NAverages = 3) were used to assess the tumor burden, and gadolinium-based contrast (Gadovist, Bayer Inc, Mississauga, Ontario, Canada) was administered either intravenously (0.1 ml/kg) or intraperitoneally (0.5 ml/kg). Intraperitoneal administration was used when venous access was compromised as well as during some of the follow-up imaging. In the latter case this was done to spare the animals unnecessary catheterizations, as catheterizations repeated at close intervals require a new insertion point each time, making the procedure progressively more difficult and leading to a greater likelihood of bruising and discomfort. Serial imaging with a delayed imaging start or longer imaging times (>20 mins) was used with intraperitoneal injections to ensure that similar contrast levels were achieved compared to intravenous administration. The animals were imaged supine, positioned on a Bruker rat brain receive surface coil to improve the image quality for assessing the tumor volume. CE-T_1_-weighted imaging was also used for the FUS + MBs treatments for treatment guidance and to assess the integrity of the BBB post-FUS + MBs. The surface receive coil was not compatible with the FUS + MBs setup and so imaging was performed using the body coil and additional averages (NAverages = 9) to improve the image quality.

### Treatment Arms

This study had three treatment arms: (1) no treatment, n = 5; (2) trastuzumab only, n = 6; (3) trastuzumab + FUS + MBs, n = 7. Of the remaining 2 animals, one did not show any tumor progression and the other developed a large subcutaneous tumor. In both cases this was attributed to an issue with the catheterization and these animals were removed from the study. Animals were randomly assigned to either group 2 or 3 upon detection of tumors via MRI. Three animals had venous access issues that prevented them from being assigned to a treatment group and so became untreated controls (Group 1), with two additional animals randomly assigned to this group. Group 2 and 3 animals received 3 weekly treatments with intravenously administered trastuzumab: an 8 mg/kg loading dose in week 1, followed by a 6 mg/kg maintenance dose in each of weeks 2 and 3. Group 2 animals received the drug alone, while Group 3 animals received concurrent FUS + MB treatments.

### Focused Ultrasound

The experimental setup for the FUS + MBs treatments is illustrated in Fig. 4C. Group 3 animals were anesthetized with isofluorane at 2% and the hair was removed from the back using an electric razor followed by depilatory cream. A 24 G angiocath was inserted into the tail vein. The animals were placed supine on a sled that could be moved between the FUS treatment platform (RK-100, FUS Instruments Inc, Toronto, Canada) and the bore of the 7 T MRI for pre and post-treatment imaging. The animals were acoustically coupled to the transducer within the treatment platform via a water bath.

Ultrasound was generated using a 75 mm diameter spherically focused lead zirconate titanate (PZT) transducer with a 60 mm focal length and a center frequency of 551.5 kHz. This frequency is more clinically relevant than that previously used^19^ and also yielded a bigger focal spot that allowed larger volumes to be covered per sonication (limit of 4 points per sonication with the FUS treatment platform). The transducer was impedance matched to 50Ω, 0° at its fundamental frequency using an external matching network and was driven using a function generator and RF amplifier. Ultrasound exposures consisted of 10 ms bursts delivered at a 1 Hz pulse repetition frequency for a total of 2 minutes. The acoustic power and the spatial and temporal peak negative pressure amplitude were measured in water using an in-house constructed radiation force measurement system and a fibre optic hydrophone (Precision Acoustics, Dorchester, UK), respectively. The FUS treatment platform allowed for mechanical scanning of the transducer and up to four foci could be target in a single treatment by interleaving the exposures. Definity microbubbles (0.02 ml/kg, Lantheus Medical Imaging, Billerica, Mass, USA) were injected intravenously via a tail vein catheter simultaneously with the start of each sonication, and a minimum of 5 minutes was allowed between each bubble injection to allow the bubbles from the prior injection to clear (Definity half-life ~ 1.3 min, product insert). A custom-built wideband receiver, similar to that described in^29^ was used to detect the bubble emissions during treatment. These signals were recorded to PC using a 14-bit scope card (ATS460, AlazarTech, Pointe-Claire, Quebec, Canada). The treatment pressure was actively controlled based on analysis of the spectral content of the recorded emissions using a method previously described for drug-delivery to the brain^30^. This approach increases the pressure for each subsequent burst until ultraharmonic signal content is detected and then drops the pressure to an effective but safe exposure level, typically 50%^30^, and maintains it for the remainder of the sonication. The mean peak pressure (±S.D.) across all treatments (158 focal spots) reached during the ramping of the exposure was 0.43 ± 0.09 MPa (steady state pressure of 0.22 ± 0.04 MPa). For one treatment, in a single animal, a hardware failure with the acoustic receiver made actively controlled exposures impossible. For this treatment a fixed acoustic pressure of 0.25 MPa was used. The reported pressure values are the estimates in water and are not de-rated for losses through the posterior bony elements of the spine and the muscle tissue. Transmission measurements were made at 14 locations in an *ex vivo* rat spinal canal using a fiber optic hydrophone (Precision Acoustics, United Kingdom). A mean transmission of 67 ± 15% was found, with a range of 45% to 92% depending on the position of the ultrasound focus with respect to the vertebrae.

Treatment targets were selected to cover the enhancing tumor region seen via MRI and treatments were repeated at different targets as necessary until full coverage of the tumor was obtained. The trastuzumab dose was injected intravenously prior to the first sonication so that the long-circulating drug would be in the circulation at the time of the ultrasound exposures. Post-treatment MRI was used to assess the integrity of the BSCB following FUS + MBs. Representative pre and post-FUS + MBs MR images are shown in Fig. 4D.

### Study Endpoints

Standard animal welfare endpoints were observed for this study, including failure to groom, >20% weight loss, vocalization, etc. Additionally, the animals were scored daily using the Basso, Beattie and Bresnahan thoracic spinal cord injury scale^31^. This 22 point scale scores hindlimb function over a 4 minute period of movement in an open field. The animals were removed from the study when the score dropped to 5 (slight movement of two hind limb joints and extensive movement of a third joint) or lower, as progression from this point to full paralysis was expected to be rapid. The rats were acclimatized for three weeks to the open field conditions at the start of the study, prior to the surgical procedures and tumor implantation.

### Histological Processing

When the study endpoints were reached the animals were sacrificed and their spines, from the incision at L4 to the top of the thoracic spine, were harvested and fixed in 10% neutral-buffered formalin. The bones were decalcified and carefully removed from the spinal cord. Following routine processing and embedding, the cords were sectioned, obtaining 5 μm thick sagittal sections at 100 μm intervals. The sections were stained with Hematoxylin and Eosin (H&E) and digitized using a Zeiss AxioImager microscope.

### Data Analysis

The MRI data were analyzed in MIPAV (https://mipav.cit.nih.gov/) and the digitized histology sections were analyzed using ImageJ (https://imagej.nih.gov/ij/index.html). The tumor areas were contoured on each image slice/histology section to estimate the total tumor volume at each imaging time-point or at the point of sacrifice. The data were compared using a one-way Analysis of Variance (ANOVA) and Tukey’s post-hoc test. As the survival time of the untreated controls was short, for later MRI time points comparisons were made between the remaining groups (trastuzumab only and trastuzumab + FUS + MBs) using a t-test with equal variance.

### Data Availability

Original data are available from the authors upon request.
